# Experiences of general practitioners participating in oncology meetings with specialists to support GP-led survivorship care; an interview study from the Netherlands

**DOI:** 10.1080/13814788.2018.1478960

**Published:** 2018-06-28

**Authors:** Anne van Leeuwen, Jan Wind, Henk van Weert, Vrony de Wolff, Kristel van Asselt

**Affiliations:** a Department of General Practice, Academic Medical Centre, University of Amsterdam, Amsterdam, The Netherlands;; b Medisch Centrum Brielle, Brielle, The Netherlands

**Keywords:** Colon cancer, GP-led survivorship care, qualitative study

## Abstract

**Background:** Due to ageing, increasing cancer incidence and improved treatment, the number of survivors of cancer increases. To overcome the growing demand for hospital care survivorship by the involvement of the general practitioner (GP) has been suggested. Dutch GPs started a project to offer survivorship care to their patients with the help of monthly oncology meetings with hospital specialists.

**Objectives:** To evaluate the experiences of GPs with monthly oncology meetings in a GP-practice to support GP-led survivorship care of colon cancer patients.

**Methods:** This is a qualitative study in primary care centres in a region in the Netherlands around one hospital. GPs were recruited from practices organizing monthly oncology meetings with hospital specialists. Ten of 15 participating GPs were interviewed until saturation. The interviews were transcribed verbatim and two independent researchers analysed the data.

**Results:** The oncology meetings and individual care plans attributed to a feeling of shared responsibility for the patients by the GP and the specialist. The meetings helped the GPs to be informed about the patients in the diagnostic and treatment phase, which was followed by a clear moment of transfer from hospital to primary care. GPs were better equipped to treat comorbidity and were more confident in providing survivorship care. Due to lack of reimbursement for survivorship care, the internal motivation of the GP must high.

**Conclusion:** The oncology meetings fulfil the need for information and communication. Close cooperation between GPs and oncology specialists appears to be an essential factor for GPs to value GP-led survivorship care positively.

KEY MESSAGES

- General practitioners feel confident to take over survivorship care in colon cancer patients.
- Monthly contact with specialists and a clear individual care plan are highly valued.
- The potential to include preventive care in cancer survivors is hardly utilized.

## Introduction

The number of new cancer diagnoses and cancer survivors is proliferating [1]. Colon cancer is one of the cancers with an increasing incidence because of ageing of the population. Moreover, the number of new patients has increased due to early detection by population screening. In the Netherlands, the population screening of colon cancer started in 2014. Because of the increased demand for hospital care, colon cancer is the candidate for alternatives of hospital-led survivorship care. After curative treatment survivorship care starts; patients are followed-up by medical specialists with the purpose of detecting recurrent disease in an early stage and to offer rehabilitation care to manage long-term side effects, including physical, functional, and psychosocial symptoms [2]. For colon cancer, asymptomatic recurrences are detected in scheduled appointments in 55% of all patients with recurrences [3].

For several hereafter-noted reasons, primary care services seem well placed to offer survivorship care [2,4]. General practitioners (GPs) often develop long-term relationships with their patients and have an understanding of the social context of a patient’s diagnosis. Therefore, they are better equipped to provide comprehensive care, including care for multimorbidity and psychosocial care [5–7]. Also, primary care services tend to focus on health promotion and health surveillance. They are easier to access than hospital services, especially in rural areas [8,9]. Lastly, GP-led survivorship care may be more cost-effective [9–11].

In the Netherlands, local GPs and oncology specialists initiated an innovative project. GPs were responsible for complete survivorship care including recurrence detections and rehabilitation. To support GPs, monthly oncology meetings were organized in one of the local primary care centres. The objective of the present study was to explore the experience of Dutch GPs with these monthly oncology meetings to support GP-led survivorship care of colon cancer patients in a qualitative design.

## Methods

### Study design

A qualitative approach was chosen to study the experiences of GPs participating in an oncology meeting in the general practice to support GP-led survivorship care for patients with colon cancer. The semi-structured interviews were conducted between November 2013 and February 2014.

### The oncology meetings

Fifteen GPs (permanent staff) participated in the project in a semi-rural region of Brielle, Rockanje and Oostvoorne, a community of around 35,000 inhabitants, 30 km west of Rotterdam, the Netherlands. From the start of the project, patients were referred to the GP after curative treatment. GPs received an individual care plan for each patient, made by the specialist. The individual care plan contained information about treatment, the follow-up protocol, and anticipated long-term side effects. In addition, the patients received the follow-up protocol. A monthly oncology meeting was held at the GP-healthcare centre (7 GPs) in Brielle, which also houses an outpatient clinic of the local hospital. In addition to the GP-healthcare centre, GP-practices (8 GPs) from villages nearby participated in the project. During the monthly oncology meeting, new patients were presented by oncology specialists who informed the GPs of the entire cancer trajectory, from diagnostics to treatment and follow-up of the patient. Also, there was the possibility for information exchange, e.g. regarding comorbidity and psychosocial context of a patient.

### Data collection

The inclusion criterion for the interview study was that the GPs cared for at least one-colon cancer patient. The interviews lasted 15–45 min (mean: 28 min). The interviews were planned 12 months after the project had started. The first author (AvL) carried out the semi-structured interviews (seven at the GP’s office and three by telephone). The interview guide can be found in Table 1. The interviews were recorded with the participants’ permission and transcribed verbatim. GPs were interviewed until saturation was reached (*n* = 10).

**Table 1.** Interview guide.

Information resources used
Value of oncology meetings
Experience with the follow-up protocol
Logistics and capacity in general practice
Suggested improvements

### Data analysis

Transcripts of the interviews were read, re-read, and checked against the original audio recordings for accuracy. Each transcript was evaluated with an experienced researcher (KvA; JW) and when necessary the focus of the subsequent interviews was slightly adapted to ensure richness in all themes to refine the findings. Open, axial, and selective coding was used for thematic data analysis. Open and axial coding involved line-by-line analysis of the transcripts by two researchers (AvL; KvA) independently. Conclusions drawn from the interviews were sent to the interviewed GPs for comment; 3 GPs responded and approved the conclusions.

## Results

The characteristics of the GPs are summarized in Table 2. GPs were responsible for 27 patients with colon cancer, varying from 1–6 patients per GP.

**Table 2.** Characteristics of participating GPs.

Gender	Age (years)	GP experience (years)	Follow-up of colon cancer patients (*n*)
Female	49	19	4
Male	62	35	6
Male	49	18	4
Female	44	14	1
Male	52	23	5
Female	34	5	1
Male	58	24	1
Female	57	29	1
Male	37	7	1
Female	31	4	3
Median	47.3	17.8	3

### GP-led survivorship care

The GPs mentioned the individual care plan and the oncology meetings as being the most essential requirements for providing survivorship care. The contact between GP and specialist improved the professional relationship and communication. According to one GP, the set-up created shared responsibility. Due to the meetings information of patients’ trajectory and the moment of transfer is clear to all involved persons.

[…] as the meetings ensure steady contact and easy accessibility to ask questions [to the specialists]. […] I think it is important for the GP’s motivation.

Different reasons were given by the GPs to look positively to the oncology meeting. Below we describe the effects of the information discussed at the oncology meeting on the relation with the patient, on the treatment of comorbidity and the confidence of the GPs.

*1. Improved relationship with the cancer patient.* In the oncology meeting specialist and GPs discuss the patient trajectory in the diagnostic and treatment phase. Therefore, GPs exactly know what happens to the patient. Most GPs thought visiting patients already during their treatment improves the relation with the patient. Subsequently, GPs believe that they can offer better guidance to patients in the survivorship phase due to an earlier established good relationship.

GP-led survivorship care helps maintain contact with the patient. Before the start of this project, a patient disappeared into secondary care and would return if he was cured or incurable. Now I follow the complete process, which allows me to develop a very different bond with the patient.

The participants also mentioned the GP’s ability to contact patients more easily and the possibility of involving the patient’s family in survivorship care, the GP often knows the patient’s relatives and they are, therefore, better informed about the patient’s social network.

Also, GPs have insight into pre-existing problems and especially psychosocial problems. Some GPs addressed that the oncology meeting was also used to exchange information about the patient’s background which could be relevant for the specialist in the diagnostic and treatment phase.

*2. Comorbidity and preventive care.* One participant mentioned the GP’s ability to focus on a patient’s comorbidity with cancer

And I think the GP, compared with the specialist, has a better picture of the patient’s comorbidity, for example, among elderly patients or diabetics. During the oncology meetings, we learn how comorbidity can affect treatment, so that GPs gain more expertise.

GP-led survivorship care offers the opportunity to provide preventive care to cancer survivors, for instance, advice about exercise, being overweight, and smoking. However, colon cancer was not considered as an opportunity to discuss lifestyle, contrary to for instance cardiovascular diseases.

I think that cessation of smoking does not match with survivorship of colon cancer. We offer that to COPD patients, not to cancer patients.

nutritional advice and suchlike… No, I will only provide this type of advice when asked. But 75% of the patients had some complaints about fatigue, so what I always actively recommend is to exercise.

*3. GP’s and patients confidence.* Because survivorship care is protocolized, most GPs did not feel a lack of survivorship expertise. Furthermore, the problems GPs encounter during survivorship care are common (e.g. diarrhoea, fatigue, fear of cancer recurrence) and patients are referred to primary care in a chronic stable state. The oncology meetings, arrangements with secondary care for prompt referral, and easy consultation with hospital staff were mentioned as a safety net.

The advantage of the specialist is that he is better informed, knowledge-wise. However, GPs get involved in a calmer phase of the disease. We do the same thing as a specialist who has outsourced the patient to his nurse practitioner. I have the idea that a GP can do the same job equally well. Such as the physical examination—I think we have just as much experience with examining the abdomen as a surgeon.

Some GPs stated that GP-led survivorship care could make patients insecure and worry about whether their GP is capable and well informed. Moreover, patients are not used to discussing results of blood tests of carcinoembryonic antigen (CEA) and ultrasound to detect early metastasizes with their GP. The GPs thought that patients would have more confidence in their GP because GPs and specialists work together and GPs receive up-to-date oncological knowledge. The primary anxiety of patients is the fear of cancer recurrence. Some GPs reported that their patients had more trust in them if they (the GP) knew the oncologist personally.

No, they are not insecure because a GP sees them, but because of fear of recurrence. That makes up a big part of the consultation.

### Drawbacks

Some GPs expressed concerns about the organization of GP-led recurrence detection. A practical problem mentioned by some GPs was how to ensure that patients were invited for a consultation in time according to the follow-up protocol. The protocol was sent to both the GP and the patient, in most GP practices the practice nurse contacted the patient in case of a missed follow-up visit.

Another drawback is the lack of structural reimbursement for survivorship care. In the Netherlands GPs are paid by health insurance companies, they make agreements about reimbursements and what kind of care GPs want to deliver. There is no special tariff for survivorship care and the consultations are reimbursed as ordinary reasons to encounter. The oncology meetings are organized at the GPs’ expense. There are concerns about the lack of special reimbursement, especially important when the number of patients eligible for survivorship care will increase.

I think the GP should receive a structural compensation for this work. After all, we are performing hospital work, which saves money. So it would be weird if we would not get compensated for that.

Four of the 10 GPs considered motivation and an interest in oncology to be vital—GPs should not be obliged to take on this role and should only do so if they want to do so.

I think you have to ask yourself if you want this and want to go for it. A GP cannot be forced to participate in oncology care. Just like performing surgical interventions in the practice, you have to have a feeling for it. Above all, one should do this only if one feels like it.

## Discussion

### Main findings

In the Netherlands, GP-led survivorship care is not part of the regular care of GPs. In this project, specialists and GPs were willing to collaborate and organize monthly oncology meetings in a primary care centre to support GP-led survivorship care. Without reimbursement, GPs and specialists themselves arranged this project. Other GPs near the health centre joined the project and the oncology meeting. These meetings fulfil several functions. GPs unanimously mentioned the meetings as being essential for the GP-led survivorship care of cancer patients because they facilitate communication with specialists and provide continuous education about treatment and comorbidity. The relationship with the patient improved due to better communication with the specialists because the GPs were informed about the whole trajectory and could plan contact moments with the patient in important periods. GPs felt confident and although the GPs were the first responsible in the survivorship phase, they felt that there was shared responsibility.

### Willingness of GPs to provide survivorship care

None of the interviewed GPs in the current study hesitated to participate in survivorship care or indicated that they doubted their abilities. They organized supporting factors, such as the oncology meetings and the individual care plan. Moreover, all GPs in the same region took part (peer persuasion) which created a positive community.

The willingness in the Netherlands to take over survivorship care is not positive in general. In an earlier Dutch study, GPs were reserved when they were asked about future developments. Wind et al. found that many GPs in the Netherlands were not confident about hypothetical future GP-led survivorship care with only a third of the participating GPs rating GP-led survivorship care positively [12]. In addition, GPs in a qualitative study argued against standardized survivorship care and emphasized their wait-and-see attitude to serve patients requests of care, rather than proactive care [13].

The willingness and barriers to take the responsibility of survivorship care by GPs is studied internationally. Several reviews summarized the role of GPs in cancer care. Lawrence et al. [14] included 35 quantitative and qualitative studies (14 from Europe) and concluded that the most mentioned barriers were lack of expertise, time and workload pressure, poor funding, lack of protocols and medicolegal risks. Additional barriers mentioned by Meiklejohn [15] were a perceived unclear role in cancer care and an unclear moment to interfere after hospital treatment. From a patient perspective, the systematic review underlined the important condition of an already existing trustful relationship with the GP.

In this project, these requirements have been met: the GPs had a longstanding relationship with their patients; there was a clear protocol, a clear moment of transfer to primary care and training by the oncology meeting.

### Communication between primary care and cancer specialists

In our study, GPs were well informed about the cancer trajectory in the hospital and felt supported by the specialist and care plan. This corresponds with recommendations from a systematic review, which addressed the communication between the primary care provider and cancer specialist [16]. The authors formulated recommendations to improve the transmural communication. Direct communication between GP and cancer specialist was described as important, and to avoid using patients for information transfer. Furthermore, the GP should schedule visits during active treatment and the cancer specialist should encourage the patient to visit the GP. Potosky et al. concluded that communication between primary and secondary care providers increases GPs’ confidence in their knowledge [17]. Also, patients seemed to value effective communication and coordination among their providers [18,19].

### Models of GP-led survivorship care

This study is an example of GP-led survivorship care, which was organized by the health professionals themselves. Several strategies to integrate cancer care in primary care were studied by Nekhlyudov and she concluded that no model fits in all circumstances [20]. O’Malley described different projects of innovators and appealed for other projects to improve cancer survivorship care in primary care [21]. Although our study is that kind of an example, we need to consider that while it may be relatively easy to prepare an individual care plan, it is more difficult to organize oncology meetings. These meetings require cooperation and close contact between primary and secondary care. This study was carried out in an area with only one local hospital. Regular meetings will be more complicated in a setting, where one general practice may cooperate with several hospitals.

### Preventive care in GP-led survivorship care

A recent study showed that survivors prefer to discuss diet and physical fitness with their GP [22]. Moreover, risk factors for colon cancer are partly similar to those for cardiovascular diseases [23]. This is not commonly recognized and is not covered in survivorship care plans. The GPs who participated in this study did not regularly offer preventive advice to their patients after cancer treatment but did continue with preventive care in the context of lifestyle programmes already started for cardiovascular, lung or diabetic care. Putting more emphasis on preventive measures during the survivorship phase may improve the efficacy of follow-up in primary care.

### Limitations and strengths

A limitation of the study is that participating GPs were primarily in favour of this project and their responses might have been susceptible to social desirability bias. Since the interviewed GPs were founders and supporters of this project, it can be imagined that they would want to present a positive image of the survivorship care that they set up. They mentioned few disadvantages. Although the GPs were interviewed some time ago, the project is still ongoing under the same circumstances; however, some reimbursement is now available.

## Conclusion

This study shows that cancer GP-led survivorship care can be managed in primary care. The GPs and specialists from this pioneer centre were able to organize survivorship care in cancer patients in a satisfying structure by monthly oncology meetings in the primary care centres.

## References

[1] SCK of KWF Kankerbestrijding Aftercare in cancer; the role of primary care. Dutch Cancer Society’s Signalling Committee on Cancer; 2011.

[2] Health Council of the Netherlands Follow-up in oncology. Identify objectives, substantiate actions. Den Haag: Health Council; 2007.

[3] Duineveld LA, van Asselt KM, Bemelman WA, et al. Symptomatic and asymptomatic colon cancer recurrence: a multicenter cohort study. Ann Fam Med. 2016;14:215–220. 27184991 10.1370/afm.1919 PMC4868559

[4] Weert van H ‘The emperor of all maladies’: towards an evidence based integrated cancer survivorship care in general practice. Eur J Gen Pract. 2016;22:69–70. 27292291 10.1080/13814788.2016.1180361

[5] Greenfield DM, Absolom K, Eiser C, et al. Follow-up care for cancer survivors: the views of clinicians. Br J Cancer. 2009;101:568–574. 19638984 10.1038/sj.bjc.6605160 PMC2736807

[6] Nekhlyudov L ‘Doc, should I see you or my oncologist?’ A primary care perspective on opportunities and challenges in providing comprehensive care for cancer survivors. J Clin Oncol. 2009;29:2424–2426. 10.1200/JCO.2008.21.4023 19332710

[7] Jefford M, Karahalios E, Pollard A, et al. Survivorship issues following treatment completion- results from focus groups with Australian cancer survivors and health professionals. J Cancer Surviv. 2008;2:20–32. 18648984 10.1007/s11764-008-0043-4

[8] Pascoe SW Psychosocial care for cancer patients in primary care? Recognition of opportunities for cancer care. Fam Pract. 2004;21:437–442. 15249534 10.1093/fampra/cmh415

[9] Lewis RA, Neal RD, Williams NH, et al. Follow-up of cancer in primary care versus secondary care: systematic review. Br J Gen Pract. 2009;59:e234–e247. 19566990 10.3399/bjgp09X453567 PMC2702037

[10] Augestad KM, Norum J, Dehof S, et al. Cost-effectiveness and quality of life in surgeon versus general practitioner-organised colon cancer surveillance: a randomised controlled trial. BMJ Open. 2013;3:e002391. 10.1136/bmjopen-2012-002391 PMC3641467 23564936

[11] Wattchow DA, Weller DP, Esterman A, et al. General practice vs surgical-based follow-up for patients with colon cancer: randomised controlled trial. Br J Cancer. 2006;94:1116–1121. 16622437 10.1038/sj.bjc.6603052 PMC2361245

[12] Wind J, Duineveld LA, van der Heijden RP, et al. Follow-up after colon cancer treatment in the Netherlands; a survey of patients, GPs, and colorectal surgeons. Eur J Surg Oncol. 2013;39:837–843. 23692700 10.1016/j.ejso.2013.04.001

[13] Geelen E, Krumeich A, Schellevis FG, et al. General practitioners’ perceptions of their role in cancer follow-up care: a qualitative study in the Netherlands. Eur J Gen Pract. 2014;20:17–24. 24576124 10.3109/13814788.2013.805408

[14] Lawrence RA, McLoone JK, Wakefield CE, et al. Primary Care Physicians’ perspectives of their role in cancer care: a systematic review. J Gen Intern Med. 2016;31:1222–1236. 27220499 10.1007/s11606-016-3746-7 PMC5023605

[15] Meiklejohn JA, Mimery A, Martin JH, et al. The role of the GP in follow-up cancer care: a systematic literature review. J Cancer Surviv. 2016;10:990–1011. 27138994 10.1007/s11764-016-0545-4

[16] Dossett LA, Hudson JN, Morris AM, et al. The primary care provider (PCP)-cancer specialist relationship: a systematic review and mixed-methods meta-synthesis. CA Cancer J Clin. 2017;67:156–169. 27727446 10.3322/caac.21385 PMC5342924

[17] Potosky AL, Han PK, Rowland J, et al. Differences between primary care physicians’ and oncologists’ knowledge, attitudes and practices regarding the care of cancer survivors. J Gen Intern Med. 2011;26:1403–1410. 21785923 10.1007/s11606-011-1808-4 PMC3235622

[18] Cheung WY, Neville BA, Earle CC Associations among cancer survivorship discussion, patient and physician expectations, and receipt of follow-up care. J Clin Oncol. 2010;28:2577–2583. 20406932 10.1200/JCO.2009.26.4549

[19] Ayanian JZ, Zaslavsky AM, Arora NK, et al. Patients’ experiences with care for lung cancer and colorectal cancer: findings from the Cancer Care Outcomes Research and Surveillance Consortium. JCO. 2010;28:4154–4161. 10.1200/JCO.2009.27.3268 PMC2953972 20713876

[20] Nekhlyudov L, O’malley DM, Hudson SV Integrating primary care providers in the care of cancer survivors: gaps in evidence and future opportunities. Lancet Oncol. 2017;18:e30–e38. 28049575 10.1016/S1470-2045(16)30570-8 PMC5553291

[21] O’Malley D, Hudson SV, Nekhlyudov L, et al. Learning the landscape: implementation challenges of primary care innovators around cancer survivorship care. J Cancer Surviv. 2017;11:13–23. 27277895 10.1007/s11764-016-0555-2 PMC5145775

[22] Huibertse LJ, VanEenbergen M, DeRooij BH, et al Cancer survivors’ preference for follow-up care providers: a cross-sectional study from the population-based PROFILES-registry. Acta Oncol. 2017;56:278–287. 28068157 10.1080/0284186X.2016.1267398

[23] Harriss DJ, Atkinson G, Batterham A, et al. Lifestyle factors and colorectal cancer risk (2): a systematic review and meta-analysis of associations with leisure-time physical activity. Colorectal Disease. 2007;11:689–701. 10.1111/j.1463-1318.2009.01767.x 19207713

